# Disease progression, hospital readmissions, and clinical outcomes for patients with steroid-refractory acute graft-versus-host disease: A multicenter, retrospective study

**DOI:** 10.1038/s41409-022-01736-0

**Published:** 2022-06-23

**Authors:** Shernan G. Holtan, Jingbo Yu, Dilan Paranagama, Jackson Tang, Hannah K. Choe, Ahmad Naim, H. Joachim Deeg, John Galvin

**Affiliations:** 1 Univeristy of Minnesota, Minneapolis, MN USA; 2 Incyte Corporation, Wilmington, DE USA; 3 Asclepius Analytics, New York, NY USA; 4 The Ohio State University, Columbus, OH USA; 5 Fred Hutchinson Cancer Research Center, Seattle, WA USA; 6 University of Illinois Cancer Center, Chicago, IL USA

**Keywords:** Graft-versus-host disease, Bone marrow transplantation

## Abstract

Acute graft-versus-host disease (GVHD) is a significant cause of morbidity and mortality following allogeneic hematopoietic cell transplantation (HCT). This analysis of 168 patients (mean age, 54.8 years) from a multicenter, retrospective chart review describes the clinical course, treatment patterns, hospitalizations, and clinical outcomes of patients aged ≥12 years who developed grades II–IV acute GVHD after their first allogeneic HCT (January 1, 2014, to June 30, 2016) and were refractory to or dependent on corticosteroids. Between diagnosis and maximum grade (median, 6.0 days), 53.6% of patients had new organ involvement, particularly lower gastrointestinal tract acute GVHD, or an increase in acute GVHD grade. Eighty-nine patients (53.0%) received additional systemic GVHD therapy (after systemic corticosteroids) within a median of 21.0 days. Hospital readmission(s) was required for 56.5% of patients within 100 days post-HCT (mean inpatient length of readmission stay, 49.5 days); 24.4% had ≥2 readmissions within 100 days post-HCT. From the date of acute GVHD diagnosis, 70.2% of patients died at a median (interquartile range) of 117.5 (49–258) days. In summary, steroid-refractory and steroid-dependent acute GVHD is associated with a rapidly worsening clinical course that leads to high readmission and mortality rates, emphasizing the need for effective and tolerable therapies.

## Introduction

Allogeneic hematopoietic cell transplantation (HCT) offers potentially curative treatment for several hematologic malignancies and nonmalignant disorders [1, 2]. More than 8 000 allogeneic HCTs are performed annually in the United States, with a trend for year-over-year increases [2]. Approximately 30–60% of allogeneic HCT recipients develop acute graft-versus-host disease (GVHD) [3–6], which has a significant impact on morbidity and mortality [7].

The standard first-line therapy for grades II to IV acute GVHD is systemic corticosteroids, although these provide effective control in only 35–60% of patients [8–10]. Patients may experience steroid dependence, typically defined as the inability to taper steroids (ie, reduce prednisone dose to <2 mg/kg/day) or the recurrence of acute GVHD in the same or new organs while tapering [11]. Acute GVHD that progresses within 3 days or fails to improve within 5–7 days of initiation of corticosteroid treatment is generally considered steroid refractory [12].

Early responses to primary acute GVHD treatment are indicative of improved long-term outcomes [13], whereas low rates of survival have been reported for patients with steroid-refractory acute GVHD [13, 14]. Previous studies, with study periods ranging from 2004 through 2015, have reported post-HCT 1-year survival rates of ≤30% for patients with steroid-refractory acute GVHD [8, 15, 16].

The objective of this analysis was to describe the clinical course, treatment patterns, outcomes, and hospital readmissions among patients with acute GVHD who were refractory to or dependent on systemic corticosteroids in a real-world setting based on findings from a multicenter retrospective chart review.

## Materials and methods

### Study design and patients

A multicenter retrospective chart review of patients who received allogeneic HCT between January 1, 2014, and June 30, 2016, (study period) was conducted at 11 US academic and community transplant centers [17]. Study sites were required to have conducted ≥50 adult allogeneic HCTs and to have diagnosed and treated ≥20 patients for acute GVHD during the study period. Institutional review board approval was obtained from participating institutions.

At each site, patients were considered eligible for inclusion if they were aged ≥12 years, had undergone their first allogeneic HCT during the study period, and had subsequently developed grades II, III, or IV acute GVHD, per the International Bone Marrow Transplant Registry Severity Index at any time during the follow-up period (ie, transplant to the end of data availability or death). Exclusion criteria included having undergone >1 allogeneic HCT, participating in a trial for GVHD prophylaxis during the study period (GVHD treatment trial permitted), having used Janus kinase inhibitors for any condition, and being unable to disclose complete GVHD-related medical history for any reason.

### Data collection

Medical records of eligible patients were reviewed by site physicians or clinical research staff. Deidentified patient data were collected through an electronic form from the date of allogeneic HCT to the most recent follow-up (data collection, March through December 2018) or death. Centers sampled patients with acute GVHD based on transplant date, beginning with the most recent transplant recipients. Data collected included patient demographics, transplant-related characteristics, acute GVHD characteristics (including diagnosis as well as grade and organs involved at diagnosis and at maximum grade), treatments received for acute GVHD, inpatient care utilization, and clinical outcomes (including acute GVHD recurrence, overall rate of infections [viral, bacterial, mycobacterial, fungal, and parasitic] and all-cause mortality).

This analysis evaluated patients who were refractory to or dependent on systemic corticosteroids. The criteria for corticosteroid dependence and refractoriness varied across participating centers. For the purposes of this analysis, corticosteroid refractory was defined as requiring the use of ≥1 additional systemic GVHD therapy. Corticosteroid dependence was defined as not being able to taper high-dose corticosteroids (≥1 mg/kg) by ≥25% or being able to taper corticosteroid dose by ≥25% but not able to taper to <10 mg/day.

### Statistical analyses

Frequencies and percentages were reported for categorical variables; mean, SD, median, and interquartile range (IQR) values were calculated for continuous variables. Mortality was calculated using Kaplan-Meier estimates. Descriptive statistics were used for consideration of numeric differences between groups of patients who were corticosteroid-refractory or -dependent; no formal statistical comparisons were performed.

## Results

### Patient demographics and clinical characteristics

The analysis included 168 patients with corticosteroid-refractory (*n* = 113) or -dependent (*n* = 55) acute GVHD (Table 1) from a cohort of 475 patients with grades II to IV acute GVHD, with data collected from 11 transplant centers (see Acknowledgements). Mean (SD) age among the 168 patients at HCT was 54.8 (12.5) years, including 1.8%, 13.7%, and 84.5% in age groups <18 years, 18–40 years, and >40 years, respectively. Most patients (63.7%) were male. The most common underlying malignancies were acute myeloid leukemia (35.1%), myelodysplastic syndrome (19.0%), and acute lymphoid leukemia (16.1%). The main stem cell source was peripheral blood (73.8%). The most commonly used transplant conditioning regimens were high-dose myeloablative regimens among patients who were steroid-refractory (58/113 [51.3%]) and reduced-intensity regimens among patients who were steroid-dependent (26/55 [47.3%]). Median (IQR) time from transplant to acute GVHD diagnosis was 30.5 (21–49) days and from acute GVHD diagnosis to death/last visit was 194 (58–720) days.

**Table 1:** Patient Demographics and Baseline Clinical Characteristics.

	Steroid-Refractory Acute GVHD (*n* = 113)	Steroid-Dependent Acute GVHD (*n* = 55)	Total Population(*N* = 168)
Age, y, mean (SD)	53.6 (13.2)	57.3 (10.5)	54.8 (12.5)
Age groups, y, *n* (%)			
<18	3 (2.7)	0	3 (1.8)
18–40	19 (16.8)	4 (7.3)	23 (13.7)
>40	91 (80.5)	51 (92.7)	142 (84.5)
Male, *n* (%)	69 (61.1)	38 (69.1)	107 (63.7)
Race, *n* (%)			
White	96 (85.0)	50 (90.9)	146 (86.9)
Black	4 (3.5)	3 (5.5)	7 (4.2)
Other	5 (4.4)	1 (1.8)	6 (3.6)
Unknown	7 (6.2)	0	7 (4.2)
Insurance status at transplant,* *n* (%)			
Private or group health insurance	77 (68.1)	38 (69.1)	115 (68.5)
Medicare	32 (28.3)	16 (29.1)	48 (28.6)
Medicaid	10 (8.8)	1 (1.8)	11 (6.5)
Other	7 (6.2)	3 (5.5)	10 (6.0)
Underlying malignancy, *n* (%)			
Acute myeloid leukemia	40 (35.4)	19 (34.5)	59 (35.1)
Myelodysplastic syndrome	22 (19.5)	10 (18.2)	32 (19.0)
Acute lymphoid leukemia	18 (15.9)	9 (16.4)	27 (16.1)
Chronic myeloid leukemia	8 (7.1)	4 (7.3)	12 (7.1)
Multiple myeloma	8 (7.1)	3 (5.5)	11 (6.5)
Non-Hodgkin lymphoma	6 (5.3)	5 (9.1)	11 (6.5)
Other	11 (9.7)	5 (9.1)	16 (9.5)
Remission status of primary disease at transplant, *n* (%)			
Complete remission	74 (65.5)	33 (60.0)	107 (63.7)
Stable disease	16 (14.2)	9 (16.4)	25 (14.9)
Partial remission	11 (9.7)	7 (12.7)	18 (10.7)
Progressive disease	5 (4.4)	4 (7.3)	9 (5.4)
Not assessed	7 (6.2)	2 (3.6)	9 (5.4)
HCT Comorbidity Index at transplant, *n* (%)			
Low risk (0)	16 (14.2)	7 (12.7)	23 (13.7)
Intermediate risk (1–2)	31 (27.4)	18 (32.7)	49 (29.2)
High risk (≥3)	63 (55.8)	26 (47.3)	89 (53.0)
Unknown	3 (2.7)	4 (7.3)	7 (4.2)
Year of transplant, *n* (%)			
2014	29 (25.7)	14 (25.5)	43 (25.6)
2015	63 (55.8)	29 (52.7)	92 (54.8)
2016	21 (18.6)	12 (21.8)	33 (19.6)
Transplant setting,^†^ *n* (%)			
Inpatient	99 (87.6)	54 (98.2)	153 (91.1)
Outpatient	11 (9.7)	1 (1.8)	12 (7.1)
Graft source, *n* (%)			
Peripheral blood	81 (71.7)	43 (78.2)	124 (73.8)
Umbilical cord blood	16 (14.2)	6 (10.9)	22 (13.1)
Bone marrow	12 (10.6)	6 (10.9)	18 (10.7)
Unknown	4 (3.5)	0	4 (2.4)
HLA donor type, *n* (%)			
Matched, unrelated	61 (54.0)	25 (45.5)	86 (51.2)
Matched, related	31 (27.4)	8 (14.5)	39 (23.2)
Mismatched, unrelated	16 (14.2)	8 (14.5)	24 (14.3)
Mismatched, related	4 (3.5)	14 (25.5)	18 (10.7)
Unknown	1 (0.9)	0	1 (0.6)
Transplant conditioning regimen, *n* (%)			
Myeloablative	58 (51.3)	14 (25.5)	72 (42.9)
Reduced intensity	27 (23.9)	26 (47.3)	53 (31.5)
Nonmyeloablative	28 (24.8)	15 (27.3)	43 (25.6)
GVHD prophylaxis therapy,^‡^ *n* (%)			
Tacrolimus-based	74 (71.8)	34 (61.8)	108 (74.0)
Methotrexate	50 (48.5)	17 (30.9)	67 (45.9)
Mycophenolate	44 (42.7)	23 (41.8)	67 (45.9)
Cyclosporine-based	28 (27.2)	7 (12.7)	35 (24.0)
Antithymocyte globulin	13 (12.6)	8 (14.5)	21 (14.4)
High-dose cyclophosphamide (posttransplant)	1 (1.0)	9 (16.4)	10 (6.8)
Sirolimus	1 (1.0)	5 (9.1)	6 (4.1)
Other	2 (1.9)	7 (12.7)	9 (6.2)
Duration of follow-up since transplant,^§^ d, mean (SD)	504.9 (469.3)	497.0 (472.0)	502.3 (468.8)

GVHD, graft-versus-host disease; HCT, hematopoietic cell transplantation; HLA, human leukocyte antigen.

*Insurance status was not available for 1 patient (steroid refractory); 18 patients (10.7%; steroid refractory, *n* = 15; steroid dependent, *n* = 3) had multiple types of insurance coverage; 1 patient (0.6%; steroid refractory) was uninsured.

^†^Transplant setting was unknown for 3 patients (steroid refractory). ^‡^Patients could receive >1 type of prophylactic therapy.

^§^Patients were followed for ≥2 years from transplant until death or end of observation, whichever occurred first.

### Disease progression

**Fig. 1:**
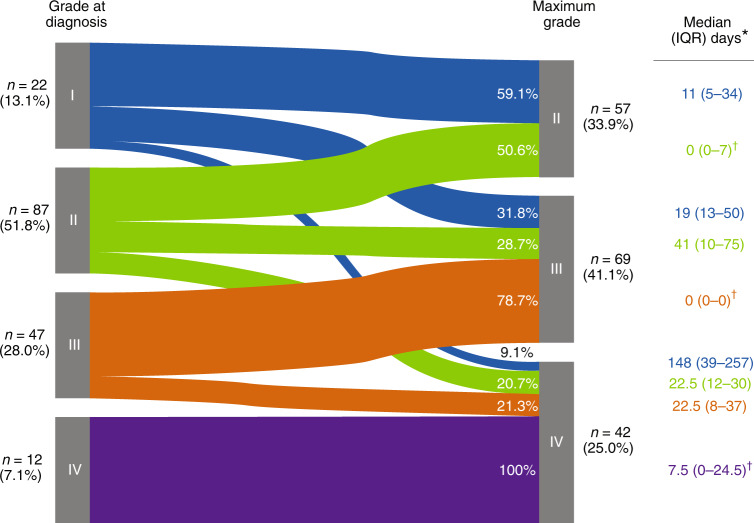
Acute GVHD severity at diagnosis and at time of maximum grade (*N* = 168). GVHD Graft-versus-host disease, IQR Interquartile range. *Time from acute GVHD diagnosis to maximum grade. ^†^Patients may remain at the same grade from diagnosis to maximum grade but progress with new organ involvement.

**Fig. 2:**
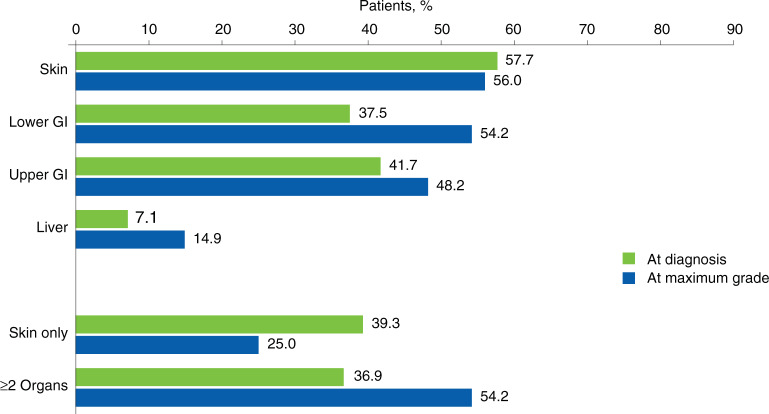
Acute GVHD organs involved at diagnosis and at time of maximum grade (*N* = 168). GI Gastrointestinal tract, GVHD Graft-versus-host disease.

At the time of acute GVHD diagnosis, most patients (109/168 [64.9%]) had grades I or II disease (Fig. 1). Almost half of patients with grade II disease (43/87 [49.4%]) progressed to a higher grade during follow-up. At the time of maximum acute GVHD grade, most patients (111/168 [66.1%]) had grade III or IV disease. Among patients who had skin-only involvement at diagnosis, 24/66 (36.4%) developed acute GVHD in other organs. During progression from acute GVHD diagnosis to maximum grade among the 168 patients, there was an increase in the proportion who had lower gastrointestinal involvement (from 37.5% to 54.2%) and ≥2 organs involved (from 36.9% to 54.2%; Fig. 2). Between the time of diagnosis and maximum acute GVHD grade, 53.6% (90/168) of patients had new organ involvement or an increase in acute GVHD grade. Median (IQR) time from acute GVHD diagnosis to maximum grade was 6.0 (0–29.5) days; patients who were steroid-refractory progressed to maximum grade over a longer period of time than those who were steroid-dependent (median, 10.0 vs 2.0 days).

### Treatment Patterns

All 168 patients received corticosteroids only as first-line therapy; 22 patients were diagnosed with grade I acute GVHD that later progressed to grades II–IV. Among 146 patients with grades II to IV acute GVHD at diagnosis, 81.5% (*n* = 119) were given systemic corticosteroids as first-line therapy; the remaining 18.5% (*n* = 27) first received topical corticosteroids. Almost half (49.3%) of the 146 patients initiated systemic corticosteroids on the day of diagnosis. The mean (SD) daily starting dose among the 119 patients with first-line systemic corticosteroid therapy was 77 (44.5) mg (0.9 [0.56] mg/kg) for prednisone and 166 (853.9) mg (1.8 [7.79] mg/kg) for methylprednisolone. During the follow-up period (time from GVHD diagnosis to end of data availability; median, 194 days), 36.3% of 168 patients with steroid-refractory or -dependent acute GVHD had an increase in steroid dose, and 87.5% were unable to taper below 10 mg/day.

**Fig. 3:**
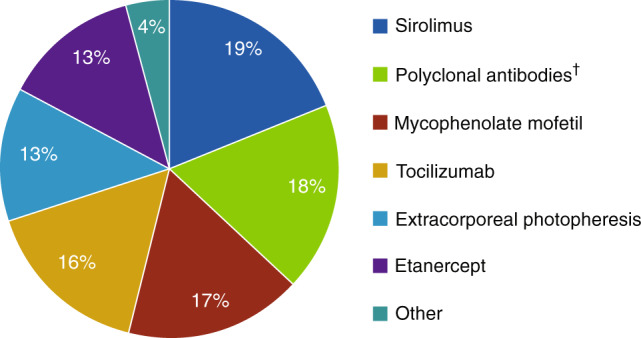
Additional systemic anti-GVHD therapy (*n* = 89). *GVHD Graft-versus-host disease. *25.8% of patients used ≥2 additional therapies. ^†^Polyclonal antibodies included antithymocyte globulin and antilymphocyte globulin.

Approximately half of patients (89/168 [53.0%]) received ≥1 line of any additional systemic GVHD therapy (Fig. 3). Of these 89 patients, 33.7% (all steroid-refractory) had an increase in corticosteroid dose before receiving additional therapy; 25.8% used ≥2 additional therapies. Median (IQR) time from corticosteroid initiation to additional therapy was 21.0 (9–41) days.

### Clinical Outcomes and Survival

Acute GVHD recurred in 70/168 patients (41.7%), including 44.2% (50/113) of patients who were steroid-refractory and 36.4% (20/55) who were steroid-dependent, and was managed by increasing the corticosteroid dose in 74.3% of all patients. Mean (SD) time from complete response to first acute GVHD recurrence was 73.6 (98.4) days; acute GVHD recurred approximately 2 weeks sooner among patients who were steroid-refractory versus steroid-dependent (mean, 68.7 vs 84.6 days). Recurrence was related to steroid dose reduction in 80.0% of cases (56/70), including patients who were steroid-refractory (42/50 [84.0%]) and steroid-dependent (14/20 [70.0%]). Approximately a quarter (45/168 [26.8%]) of patients later developed chronic GVHD (similar for both groups of patients). Among patients who developed acute GVHD during their initial HCT hospital stay, median (IQR) length of stay was longer for patients with grades III/IV acute GVHD (*n* = 16; 61.5 [51–83] days) compared with those who developed grade II acute GVHD (*n* = 30; 35 [33–44] days). Hospital readmission(s) were required by 56.5% (95/168) of patients through 100 days post-HCT (mean inpatient length of stay upon readmission, 49.5 days); the mean (SD) number of hospital readmissions per patient was 1.6 (0.8), and 24.4% (41/168) had ≥2 readmissions. Primary reasons for hospital readmission among patients who were steroid-refractory and steroid-dependent, respectively, were acute GVHD (54/113 [53.5%] and 20/55 [39.4%]) and infection (14/113 [13.9%] and 7/55 [25.2%]). Half of patients (85/168 [50.6%]) experienced ≥1 infection that was confirmed and required treatment between diagnosis of acute GVHD and 100 days post-HCT. Most infections were viral (*n* = 69) and included cytomegalovirus (reactivation without disease [*n* = 33], disease [*n* = 15], recurrent disease [*n* = 4]), Epstein-Barr virus (*n* = 7), herpes simplex virus (*n* = 1), varicella-zoster virus (*n* = 1), and other viruses (*n* = 25). Bacterial (*n* = 40), fungal (*n* = 8), and mycobacterial (*n* = 1) infections were also observed. During follow-up (median, 194 days), 20.8% of patients (35/168) had a relapse of their underlying malignancy. Of the 118 deaths that occurred during follow up, 79.7% (94/118) were due to transplant-related mortality. Relapse was less common among patients who were steroid-refractory (15/113 [13.3%]) than steroid-dependent (20/55 [36.4%]).

**Fig. 4:**
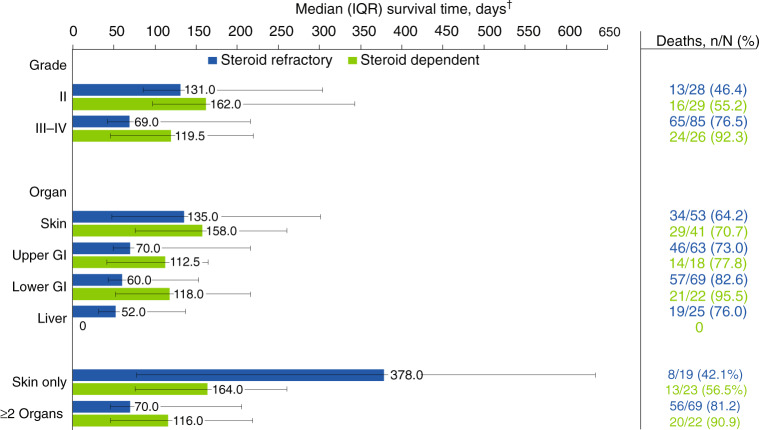
Patient deaths by acute GVHD severity and organs* involved at time of maximum grade. GI Gastrointestinal tract, GVHD Graft-versus-host disease, IQR Interquartile range. *Organ stage 1–4 was considered organ involvement; patients could have multiple organs involved. ^†^Time from acute GVHD diagnosis to death.

From the date of acute GVHD diagnosis, 70.2% of patients (118/168) died at a median (IQR) of 117.5 (49–258) days; patients who were steroid-refractory died approximately 6 weeks earlier than those who were steroid-dependent (median, 83.0 vs 128.5 days). Known causes of death in ≥2% of the 118 patients who died were primary disease (20.3%), acute GVHD (21.2%), infection (12.7%), organ failure (6.8%), and chronic GVHD (2.5%); the leading cause of death was acute GVHD among patients who were steroid-refractory (23/78 [29.5%]) and primary disease among patients who were steroid-dependent (15/40 [37.5%]). Mortality data by acute GVHD grade and organ involvement are shown in Fig. 4. Among those with acute GVHD progression, 82.2% (74/90) of patients died at a median (IQR) of 116.0 (49–223) days. Of 111 patients with maximum grades III/IV acute GVHD, 80.2% died at a median (IQR) of 80.0 (42–216) days. Among 91 patients with lower gastrointestinal involvement at maximum grade acute GVHD, 85.7% died at a median (IQR) of 74.0 (44–174) days.

## Discussion

Despite the use of systemic treatment(s) and recent advances in supportive care and availability of novel investigational agents, the majority of patients with corticosteroid-refractory or -dependent acute GVHD assessed in this large, multicenter, retrospective chart review developed severe (grades III/IV) disease. Additionally, many patients experienced rapid disease progression, with particularly high rates of progression observed in the lower gastrointestinal tract (from 38% with involvement at diagnosis to 54% at maximum grade), highlighting the challenges in treating patients with lower gastrointestinal acute GVHD involvement. A high rate of mortality was observed in patients with steroid-refractory and steroid-dependent disease. The mortality rate of approximately 70% over a median of 117.5 days since acute GVHD diagnosis in the present study is comparable to other real-world data [15, 18, 19], suggesting that more successful strategies are needed for prevention of steroid-refractory and -dependent acute GVHD and that additional second-line and later treatment options are needed for patients who have progressed on steroids.

Higher and cumulative steroid doses are associated with an increased incidence of infection [20]. Despite this, 36% of patients included in these analyses had an increase in steroid dose during follow-up, and 88% were unable to taper below 10 mg/day. It follows that half of patients experienced infection between acute GVHD diagnosis and 100 days post-HCT, and 13% of deaths were attributed to infections. Although there is no standard second-line treatment for acute GVHD [21, 22], steroid-refractory acute GVHD is typically diagnosed within 7 days of first-line therapy [12, 22]. In these analyses, median time from systemic corticosteroid initiation to additional therapy was 21 days, and 42% of 89 patients who received ≥1 line of any additional systemic GVHD therapy first had an increase in corticosteroid dose. These data suggest that second-line therapies have delayed initiation. Furthermore, only half of the patients (89/168) included in this study received additional therapy. Treatment patterns were at the discretion of physicians, indicating that clinicians may not have perceived any benefit of additional therapy, again highlighting the need for more effective second-line treatment strategies. In May 2019, ruxolitinib, a Janus kinase (JAK) 1/JAK2 inhibitor, became the first US Food and Drug Administration–approved treatment for steroid-refractory acute GVHD in patients ≥12 years old [23]. It would be of interest to re-evaluate clinical management practices of steroid-refractory and -dependent acute GVHD from June 2019 to evaluate management and outcomes after ruxolitinib approval.

In the current analyses, more than half of the patients (56.5%) required hospital readmission with an extended length of stay. In a retrospective review of a hospital discharge database, high hospital readmission rates (77.2%) were reported for patients with high-risk or steroid-refractory acute GVHD within 100 days post-HCT [14]. Although not comparable, owing in part to differences in study design, these findings suggest that hospital readmission rates remain high for patients with steroid-refractory acute GVHD, further supporting that disease management has not yet been optimized. Effective treatments that block the major pathogenic pathways and provide rapid control of disease may help with reducing frequency and length of hospitalizations.

Limitations to this study include the retrospective nature of the analysis. Data collection was limited by available information in medical charts; treatment decisions were at the discretion of physicians from 11 different centers. Furthermore, results may not be generalizable to patients beyond those with acute GVHD who were refractory to or dependent on corticosteroids per the definitions used in this analysis. Finally, the number of patients who were steroid-dependent was small, limiting comparison with patients who were steroid-refractory.

In conclusion, a rapidly worsening clinical course and high mortality rate was observed in real-world patients with steroid-refractory and steroid-dependent acute GVHD from 11 US centers. These findings further emphasize the need for therapies that effectively prevent or reverse disease progression.

## Data Availability

Access to individual patient-level data is not available for this study. Information on Incyte’s clinical trial data sharing policy and instructions for submitting clinical trial data requests are available at: https://www.incyte.com/Portals/0/Assets/Compliance%20and%20Transparency/clinical-trial-data-sharing.pdf?ver=2020-05-21-132838-960.
